# Hypoxic Wharton's Jelly Stem Cell Conditioned Medium Induces Immunogenic Cell Death in Lymphoma Cells

**DOI:** 10.1155/2020/4670948

**Published:** 2020-04-20

**Authors:** Hao Daniel Lin, Chui-Yee Fong, Arijit Biswas, Ariff Bongso

**Affiliations:** Department of Obstetrics and Gynaecology, Yong Loo Lin School of Medicine, National University Health System, National University of Singapore, Singapore 119228

## Abstract

Mesenchymal stem cells from Wharton's jelly of the human umbilical cord (hWJSCs), and the conditioned medium (hWJSC-CM) prepared from them, were shown to be tumoricidal on many cancers. However, these tumoricidal effects were observed in hWJSCs grown under normoxic conditions of 21% oxygen in the laboratory. Since oxygen concentrations in the stem cell niche or physiological microenvironment are hypoxic and help to maintain stemness properties, the objective of this work was to evaluate whether there were differences in the tumoricidal properties of hWJSC-CM grown in 21% O_2_ (normoxic) or 5% O_2_ (hypoxic) environments. The results showed that hWJSCs grown under normoxic or hypoxic conditions showed no distinct morphological differences in culture and remained positive in trilineage differentiation into adipocytes, osteocytes, and chondrocytes. Hypoxic hWJSCs expressed the mesenchymal stem cell surface markers CD105, CD90, CD73, CD146, and CD108 similar to normoxic hWJSCs but were negative for the hematopoietic markers CD14, CD19, CD34, CD45, CD117, and HLA-DR. Hypoxic hWJSC-CM produced a significantly greater reduction in cell viability and a significantly greater increase in apoptosis, oxidative stress, and lipid peroxidation in human lymphoma cells compared to normoxic hWJSC-CM. Hypoxic hWJSC-CM also produced significantly greater expression of immunogenic cell death (ICD) hallmarks such as surface-bound calreticulin, HSP70, HSP90, and high mobility group binding 1 proteins and significantly decreased expression of the defense molecules CD47 and PD-L1. This study showed that the tumoricidal effect of hypoxic hWJSC-CM was superior to normoxic hWJSC-CM and should be the preferred choice of preparing hWJSC-CM for the induction of ICD on lymphoma cells.

## 1. Introduction

Primitive populations of mesenchymal stems cells have been derived from the gelatinous connective tissue matrix (Wharton's jelly) of the human umbilical cord (hWJSCs) [1, 2]. These hWJSCs originate from the aorta-gonad-mesonephros and through their movement finally come to reside in Wharton's jelly during early human development [3]. They can be harvested in large numbers, can proliferate rapidly, and have been widely used in the clinic to treat a variety of diseases as they do not form tumors and have high tolerance in transplantation settings [4, 5].

These hWJSCs possess tumoricidal properties. We and others have reported that hWJSCs and hWJSC-CM attenuated or abolished various carcinomas of the breast, bone, bile ducts, and bladder [6–15]. It was also reported that stem cells from the rat umbilical cord matrix induced abolishment of tumors of the mammary gland in the rat with no resulting metastases when injected intratumorally [8]. Unengineered hWJSCs homed into and reduced the tumor burden in human breast carcinomas xenografted in the rat when injected intravenously [6]. hWJSCs also stopped the proliferation of breast cancer cells by secreting dickkopf and suppressing the Wnt pathway in xenograft mice [11]. Some research groups have shown that hWJSC-CM or microvesicles derived from hWJSCs inhibited phosphoinositide 3-kinase, Akt, and Wnt/B-catenin signalling in bile duct or urinary tract cancer cells, respectively, to stop their growth [15, 16].

hWJSCs were also shown to release many molecules like IL-6, IL-8, and MCP-1 [17] that are involved in producing DAMPs on cancer cells and modulate the immune response in xenograft animal models of cancer [11]. hWJSC-CM have been shown to induce immunogenic cell death in lymphoma cells [18]. The lymphoma cells treated with hWJSC-CM undergo immunogenic cell death and exhibited “find-me/eat-me”-danger-associated molecular pattern (DAMP) signals such as the surface bound calreticulin (ecto-CRT), ecto-Hsp70 and ecto-Hsp90, adenosine thiophosphate, and HMGB1 and the downregulation of PD-L1 and CD47 [18–20].

All the above studies used mesenchymal stromal or stem cells grown under normoxic conditions. However, it has been shown that the oxygen tension in the umbilical cord in which hWJSCs reside is between 1.8% and 8% (rarely exceeding 5%) [21, 22]. Oxygen levels in the stem cell niches or physiological microenvironment of the umbilical cord play a vital role in maintaining their stemness properties. Therefore, culturing of MSCs under normoxic conditions does not mimic the physiological microenvironment, thus promoting replicative stress, genetic instability, senescence, and cell death. More crucially, hypoxic preconditioning of MSCs prior to administration showed increased therapeutic potential in treating cardiac ischemia, critical limb ischemia, traumatic brain injury, and liver regeneration [23]. MSCs grown under hypoxic conditions secreted more tropomyosins and other proteins like endoplasmin precursor, annexin A1, transgelin, and nucleoside diphosphate kinase B that facilitated cardiac repair and prevented the induction of ventricular arrhythmias after myocardial infarction in the rat model [24]. Likewise, adipose-derived stem cells (ASCs) grown under hypoxic conditions secreted more anti-inflammatory and regenerative mediators like IL-6, tumor necrosis factor a, hepatocyte growth factor, and vascular endothelial growth factor that promote liver regeneration and function as compared to normoxic ASCs [25].

Bone marrow MSCs (BM-MSCs) expanded under hypoxic conditions upregulated chemokine receptors like CXCR4 and CX3CR1 and were able to enhance migration to the tumor [26]. The same study also indicated that hypoxic MSCs are more superior as carriers that deliver anticancer drugs to target tumors than normoxic MSCs. Likewise, another group also reported that adult human adipose MSCs (ASCs) cultured under hypoxic conditions had greater motility and greater homing ability to glioblastomas both *in vitro* and *in vivo* as compared to normoxic-cultured hAd-MSCs [27]. Both papers suggested that MSCs cultured close to their hypoxic physiological oxygen tension levels had higher survival, better cancer tropism, and greater anticancer effects when transplanted into *in vivo* animal models. Given this background, we sought to comparatively evaluate the tumoricidal effects of hWJSC-CM grown under hypoxic and normoxic conditions on human lymphoma cells.

## 2. Materials and Methods

### 2.1. Cell Culture

#### 2.1.1. Human Wharton's Jelly Stem Cells

Human Wharton's jelly stem cell (hWJSC) lines were obtained from human umbilical cords. Written informed patient consent by the patients themselves and approval from the Singapore Ministry of Health Institutional Domain Specific Review Board (DSRB) were obtained for this study. hWJSCs were cultured in hWJSC medium comprising of 80% DMEM, 20% fetal bovine serum (FBS) (GE Healthcare Life Sciences, Utah, USA), 1% nonessential amino acids, 2 mM L-glutamine, 0.1 mM *β*-mercaptoethanol, 1% insulin-transferrin-selenium, antibiotic/antimycotic mixture (Thermo Scientific, Rochester, NY), and 16 ng/ml basic fibroblast growth factor (Millipore Bioscience, Temecula, CA). The hWJSCs were cultured in 5% CO_2_ for normoxic (21% O_2_) and hypoxic (10% O_2_ or 5% O_2_ level) conditions using a hypoxic incubator (Heracell 240i, Thermo Scientific).

#### 2.1.2. Human Lymphoma Cells

The use of commercial human lymphoma cell lines (Ramos, CRL-1596) (American Type Culture Collection, Rockville, MD, USA) for this study was approved by the National University of Singapore Institutional Review Board (NUS-IRB). The lymphoma cell lines were cultured in lymphoma medium consisting of RPMI (Thermo Scientific) supplemented with 10% heat inactivated FBS (HI-FBS) (GE Healthcare Life Sciences) and 1% antibiotic/antimycotic mixture (Thermo Scientific).

#### 2.1.3. Human Skin Fibroblasts (CCD112sk)

The use of commercial human skin fibroblasts (CCD112sk, CRL-2429) (ATCC) for this study was approved by the National University of Singapore Institutional Review Board (NUS-IRB). The skin fibroblasts were cultured in DMEM-high glucose (Thermo Scientific) supplemented with 10% FBS (GE Healthcare Life Sciences), 2 mM L-glutamine (Thermo Scientific), and 1% antibiotic/antimycotic mixture (Thermo Scientific).

### 2.2. Characterization of Normoxic and Hypoxic hWJSCs

#### 2.2.1. Plastic Adherence of Cells

hWJSCs were grown under normoxic (21% O_2_) and two hypoxic conditions (10% or 5% O_2_). The cells were monitored and imaged with a phase contrast microscope (Nikon Instruments, Tokyo, Japan).

#### 2.2.2. Cell Surface Markers

The cells grown under normoxic (21% O_2_) and hypoxic (10% or 5% O_2_) conditions were first collected using trypsin (TrypLE™ Express, Thermo Scientific) for 3-5 mins at 37°C in a 5% CO_2_ in air, washed in phosphate-buffered saline (PBS), and blocked with 10% normal goat serum (NGS) (Thermo Scientific) for 10 mins to prevent nonspecific binding. The cells were then incubated with mouse monoclonal primary antibodies for a series of CD markers—CD14, CD19, CD24, CD29, CD34, CD40, CD44, CD45, CD49d, CD73, CD90, CD105, CD117, CD140b, CD146, CD271, HLA-DR, and CD108-PE (1 : 100) (BioLegend, San Diego, CA)—for 30 mins. This was followed by incubation with Alexa Fluor®488 (1 : 500) goat anti-mouse secondary antibody (Thermo Scientific) for 30 mins. The cells were finally washed in PBS, resuspended in 10% NGS, filtered using a 40 *μ*m nylon strainer (BD) to remove any cell clumps, and analyzed using a CyAn™ ADP Analyzer (Beckman Coulter, Fullerton, CA).

#### 2.2.3. Multipotent Differentiation of Normoxic and Hypoxic hWJSCs

Cells were seeded (10 × 10^4^ cells/dish) into 6-well tissue culture plates and incubated at 37°C in a 5% CO_2_ atmosphere for 24 hr to allow for cell attachment under normoxic and hypoxic conditions. For adipogenic differentiation, the medium was changed to adipogenic induction medium containing DMEM (Thermo Scientific) supplemented with 10% FBS, 1% penicillin/streptomycin, 0.01 mg/ml insulin (Thermo Scientific), 1 *μ*M dexamethasone (Sigma), 0.5 mM 3-isobutyl-1-methyl-xanthine (IBMX) (Sigma), and 0.2 mM indomethacine (Sigma). The cells were cultured for 21 days, with fresh medium changes twice a week. The cells were then fixed with 4% paraformaldehyde for 10 mins, rinsed with PBS, and postfixed with 60% isopropanol for 5 mins. The cells were stained with Oil Red O for 5 mins, rinsed with distilled water, and counterstained with hematoxylin (Sigma).

For osteogenic differentiation, the medium was changed to osteogenic induction medium containing DMEM medium (Thermo Scientific) supplemented with 5% FBS, 0.17 mM L-ascorbic-acid (Sigma, St. Louis, MO), 100 nM dexamethasone, 1% penicillin/streptomycin, and 10 mM *β*-glycerophosphate (Sigma). The cells were cultured for 21 days with fresh changes of medium twice a week. Osteogenic mineralization was then evaluated by Von Kossa staining. Briefly, the cells were rinsed with PBS and fixed in 4% paraformaldehyde solution (Sigma) for 10 mins at room temperature. They were then washed with distilled water and stained in 1% silver nitrate solution (Sigma) under UV light for 60 mins. The cells were then counterstained with 1% Nuclear Fast Red (Sigma) for 5 mins.

For chondrogenic differentiation, the medium was changed to chondrogenic induction medium containing DMEM medium (Thermo Scientific) supplemented with 1% penicillin/streptomycin, 1% insulin-transferrin-selenium (ITS), 0.17 mM L-ascorbic-acid, 100 nM dexamethasone, 1 mM sodium pyruvate, 0.35 mM proline, and 10 ng/ml transforming growth factor beta-3 (TGF*β*-3) (Sigma). The cells were cultured for 21 days with fresh changes of medium twice weekly. The cells were fixed in 4% paraformaldehyde for 30 mins and then stained with 0.5% Alcian blue (Sigma) for 30 mins at room temperature, rinsed with tap water, and then counterstained with 0.1% Nuclear Fast Red (Sigma) for 5 mins. The stained cells were subsequently visualized and imaged using bright field optics (Nikon Instrument).

### 2.3. Preparation of Wharton's Jelly Stem Cell Conditioned Medium (hWJSC-CM)

hWJSC-CM was prepared according to our previously described reports [9, 10]. Briefly, hWJSCs at early passages (4P-6P) were first cultured in hWJSC medium to 70% confluency in normoxic and hypoxic conditions. The medium was replaced with a serum-free basal RPMI medium (Biowest) supplemented with 1% L-glutamine and antibiotic-antimycotic mixture (Thermo Scientific). After 48 hr, the hWJSC-CM was collected, centrifuged, and filtered using 0.22 *μ*M filter (Millipore) and stored at -80°C till further usage.

### 2.4. Exposure of Normoxic and Hypoxic hWJSC-CM on Lymphoma Cells

Lymphoma and other cell lines (fibroblast and hWJSC) were initially grown using the culture conditions recommended in published protocols to ensure that proper growth of the cells took place. Later, before the actual experiments were started to evaluate the effects of hWJSC-CM or fibroblast-CM, all cell lines (lymphoma and fibroblast) were grown in media supplemented with heat-inactivated fetal serum (HI-FBS). Thus, culture conditions were standardized. HI-FBS was used in all experiments for the experimental and control cell lines in all subsequent assays.

Ramos lymphoma cells (1 × 10^5^) were exposed to normoxic and hypoxic hWJSC-CM supplemented with 10% HI-FBS (Thermo Scientific) for 48 hr at 37°C in a 5% CO_2_. The cells were then subjected to a series of evaluation parameters as described in our previously published article [18]. The cells will be tested for cell viability (MTT assay), annexin V/propidium iodide (PI) analysis, mitochondrial membrane potential, mitochondrial superoxide, lipid peroxidation, apoptosis (caspases 3, 8, and 9), CD47, PD-L1 surface marker expression, and immunogenic cell death DAMP (ecto-CRT, ecto-Hsp70, ecto-Hsp90, ATP, and HMGB1 secretions).

### 2.5. MTS Assay

For cell viability analysis, an MTS assay was performed using the Promega CellTiter 96 AQueous One Solution Cell Proliferation Assay kit (Promega Corporation, Wisconsin, USA) based on the manufacturer's instructions. Briefly, 50 *μ*l of MTS reagent was added to the respective Ramos lymphoma cell medium (500 *μ*l) and incubated for about 4 hr at 37°C in a 5% CO_2_ in air atmosphere. Absorbance reading at 490 nm using a spectrophotometer ELISA reader was taken (mQuant; BioTek, Winooski, VT, USA).

### 2.6. Annexin V/Propidium Iodide Assay

The samples from each experimental group were collected, washed with PBS, centrifuged, and then resuspended in 500 *μ*l of 1x annexin V binding buffer (Thermo Scientific). The cells were then stained with 1 *μ*l of annexin V-FITC (Thermo Scientific) and 0.5 *μ*l of propidium iodide (PI, Thermo Scientific) at room temperature for 15 mins. The samples were then filtered with a 40 *μ*m nylon strainer and analyzed using a CyAn™ ADP analyzer (Beckman Coulter).

### 2.7. Functional Caspase 3/7, Caspase 8, and Caspase 9 Activity Analysis

The treated and control cells were collected and analyzed for caspase 3, 8, and 9 activities using the CellEvent™ Caspase-3/7 Green Detection Reagent, Vybrant® FAM Caspase-8 Assay kits (Thermo Scientific), and Caspase 9 (active) FITC Staining kit (Abcam, Cambridge, UK). Briefly, for caspase 3, the cells were incubated with 500 *μ*l of culture medium containing 0.125 *μ*l of the 2.0 mM CellEvent™ Caspase-3/7 Green Detection Reagent at 37°C for 30 mins. For caspase 8, the cells were resuspended with 300 *μ*l of culture medium containing 10 *μ*l of 30x FLICA at 37°C for 60 mins. For caspase 9, the cells were incubated with 300 *μ*l of culture medium containing 1 *μ*l of 30x FITC-LEHD-FMK at 37°C for 60 mins. The stained cells were washed, filtered with a 40 *μ*m strainer, and analyzed using a CyAn™ ADP analyzer (Beckman Coulter).

### 2.8. Mitochondrial Membrane Potential Assay (Δ*ψ*)

The mitochondrial membrane potential (Δ*ψ*) of the treated and control cells was collected and analyzed using the MitoTracker® Red CMXRos kit (Thermo Scientific). Briefly, the cells were incubated with 500 *μ*l of culture medium containing 1 *μ*l of 1 mM MitoTracker® Red CMXRos stock solution (Thermo Scientific) at 37°C for 30 mins. The cells were filtered with a 40 *μ*m strainer and analyzed using a CyAn™ ADP analyzer (Beckman Coulter).

### 2.9. Mitochondrial Superoxide Assay

The treated and control cells were collected and analyzed for mitochondrial superoxide using the MitoSOX™ Red mitochondrial superoxide indicator kit (Invitrogen). Briefly, the cells were incubated with 100 *μ*l of culture medium containing 0.1 *μ*l of 5 mM MitoSOX™ working solution at 37°C for 30 mins. The cells were then washed, filtered with a 40 *μ*m strainer, and analyzed using the CyAn™ ADP analyzer (Beckman Coulter).

### 2.10. Lipid Peroxidation Analysis

The treated and control cells were collected and stained using the Image-iT® Lipid Peroxidation kit based on the manufacturer's instructions (Invitrogen). Briefly, the cells were stained with 10 mM Image-iT® Lipid Peroxidation Sensor (Component A) for 30 mins at 37°C. The cells were then washed, filtered with a 40 *μ*m strainer, and analyzed using a BD LSRFortessa™ analyzer (BD Bioscience, Heidelberg, Germany) with excitation/emission filters of 581/591 nm and 488/510 nm.

### 2.11. Danger-Associated Molecular Pattern (DAMP) Analysis

#### 2.11.1. Danger-Associated Molecular Patterns (DAMPs): CRT, Hsp90, and Hsp70

The treated and control cells were collected and blocked with 10% NGS on ice for 10 mins. The cells were then incubated with primary anti-human CRT, Hsp90, and Hsp70 antibodies (1 : 500) (Abcam) for 30 mins on ice followed by secondary goat anti-mouse IgG (H+L) Alexa Fluor 488 antibodies (1 : 500) (Thermo Scientific) for 30 mins on ice in the dark. The cells were washed with PBS, resuspended in cold 10% NGS, filtered with a 40 *μ*m strainer, and analyzed using a CyAn™ ADP analyzer (Beckman Coulter, Fullerton, CA, USA).

#### 2.11.2. Extracellular ATP Concentration

The culture media from the treated and control cells were collected and stored at -80°C. The extracellular ATP concentration was determined using an ATP assay kit (Abcam) based on the manufacturer's instructions. Briefly, 50 *μ*l of supernatant from the treatment and control arms was mixed with 50 *μ*l of ATP reaction mix containing 45.8 *μ*l ATP Assay Buffer, 0.2 *μ*l ATP Probe, 2 *μ*l ATP Converter, and 2 *μ*l Developer Mix. The mixtures were incubated at room temperature for 30 mins in the dark. Absorbance at 570 nm was measured using a spectrophotometer microplate reader (mQuant; BioTek, Winooski, VT, USA).

#### 2.11.3. Extracellular HMGB1 Concentration

The culture media in which the treated and control cells were grown were collected and stored at -80°C. The extracellular HMGB1 concentration was determined using an ELISA assay kit (Uscn Life Science Inc., Wuhan, China) based on the manufacturer's instructions. Briefly, 100 *μ*l of supernatant from the treatment and control arms, diluted standards, and blanks was added into the precoated 96-well strip plate for 2 hr at 37°C. After removal of the liquid, 100 *μ*l of Detection Reagent A was added into each well and incubated for 1 hr at 37°C. The wells were then washed with 350 *μ*l of 1x Wash Solution 3 times and blotted dry on absorbent paper. 100 *μ*l of Detection Reagent B was then added and incubated for 30 mins at 37°C. The wells were then washed with 350 *μ*l of 1x Wash Solution 5 times and blotted dry on absorbent paper. 90 *μ*l of Substrate Solution was then added and incubated for 20 mins at 37°C in the dark. Lastly, 50 *μ*l of Stop Solution was added into each well, and HMBG1 levels were measured at 450 nm using a spectrophotometer microplate reader (mQuant; BioTek, Winooski, VT, USA).

#### 2.11.4. CD Marker Analysis

The treated and control cells were collected, washed once with PBS, centrifuged, and then blocked with 10% NGS on ice for 10 mins. The cells were then incubated with primary anti-human CD47 antibody (1 : 500) (BioLegend, San Diego, CA) and PD-L1 (CD274) (1 : 500) (BioLegend) on ice for 30 mins followed by secondary goat anti-mouse IgG (H+L) Alexa Fluor 488 antibody (1 : 500) (Invitrogen) for 30 mins on ice in the dark. The cells were washed with PBS, resuspended in cold 10% NGS, filtered with a 40 *μ*m strainer, and analyzed with a BD LSRFortessa™ analyzer (BD Bioscience, Heidelberg, Germany).

### 2.12. Statistical Analysis

For the experiment on the surface markers of normoxic and hypoxic hWJSCs, the results were presented as the mean ± SEM (%) and one-way ANOVA with Bonferroni's multiple comparison post hoc analysis (SPSS Statistics v 17.0 software package) (SPSS Inc., IL) between 3 groups was used to calculate any statistically significant differences in the surface markers between normoxic and hypoxic hWJSCs. For all the other experiments, the results were expressed as the mean ± SEM and statistical significance between the control and hWJSC-CM collected under hypoxic and normoxic groups for lymphoma cells was calculated using one-way ANOVA with Bonferroni's multiple comparison post hoc analysis (SPSS Statistics) between 4 groups. The *p* value of <0.05 was considered as statistically significant.

## 3. Results

### 3.1. hWJSC Surface Marker Analysis

Cell surface marker analysis for MSC CD markers using flow cytometry showed that hypoxic and normoxic hWJSCs were positive for CD105, CD90, CD73, CD146, and CD108 and did not express CD34, CD45, CD14, CD19, CD117, and HLA-DR markers (Table 1). The percentage of CD surface markers between normoxic and hypoxic hWJSCs was not significantly different.

The expression of the cell surface marker (mean fluorescence index) value showed that there were significant higher expressions of CD105, CD90, CD73, CD146, and CD108 markers in hypoxic hWJSCs (10% O_2_ and 5% O_2_) as compared to normoxic hWJSCs. Hypoxic hWJSCs (5% O_2_) showed the highest expression level of CD105, CD90, CD73, CD146, and CD108 markers as compared to normoxic hWJSCs (21% O_2_) and hypoxic hWJSCs (10% O_2_) (Table 2). There was a significantly higher expression of CD34 marker in hypoxic hWJSCs (5% O_2_) compared to normoxic hWJSCs (21% O_2_) and hypoxic hWJSCs (10% O_2_). There was a significantly higher expression of CD117 in hypoxic hWJSCs (10% O_2_) compared to normoxic hWJSCs (21% O_2_) and hypoxic hWJSCs (5% O_2_) (Table 2).

### 3.2. Viabilities of Lymphoma Cells when Treated with Normoxic or Hypoxic hWJSC-CM

**Figure 1:**
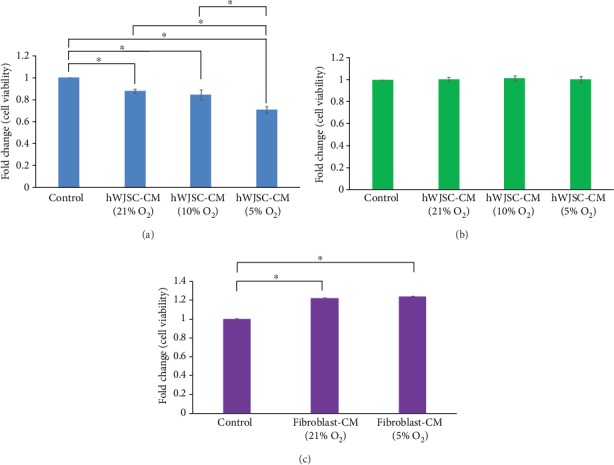
Cell viability (MTS assay) of human lymphoma and control CCD112sk cells exposed to normoxic and hypoxic hWJSC-CM for 48 h. (a) Note significant decreases in cell viability of lymphoma cells after treatment with normoxic or hypoxic hWJSC-CM as compared to controls. Treatment with hWJSC-CM collected under 5% oxygen conditions showed the greatest decrease of lymphoma cell viability. (b) Note no significant changes to the cell viability of the control cells (skin fibroblasts, CCD112sk) after treatment with hWJSC-CM. (c) Note significant increases in cell viability of lymphoma cells after treatment with normoxic or hypoxic fibroblast-CM as compared to controls. All values are expressed as the mean ± SEM of 3 biological samples with 3 replicates for each sample. ^∗^*p* < 0.05.

Lymphoma cells exposed to hypoxic and normoxic hWJSC-CM over 48 h showed significant reductions in cell viabilities compared to controls. Hypoxic hWJSC-CM (5% O_2_) induced the greatest reduction in cell viability, and the cell viability of hypoxic hWJSC-CM (5% O_2_) was significantly lower than normoxic hWJSC-CM (21% O_2_) and hypoxic hWJSC-CM (10% O_2_) (Figure 1(a)). The fold changes in cell viabilities as compared to controls were hWJSC-CM (21% O_2_): 0.88 ± 0.02, hWJSC-CM (10% O_2_): 0.84 ± 0.05, and hWJSC-CM (5% O_2_): 0.71 ± 0.03. There were no significant differences in cell viabilities for normal skin fibroblast cells when exposed to normoxic or hypoxic hWJSC-CM over 48 h (Figure 1(b)). The fold changes in cell viabilities as compared to controls were hWJSC-CM (21% O_2_): 1.00 ± 0.02, hWJSC-CM (10% O_2_): 1.00 ± 0.03, and hWJSC-CM (5% O_2_): 1.00 ± 0.03 (Figure 1(b)). Lymphoma cells exposed to hypoxic and normoxic fibroblast-CM over 48 h showed significant increases in cell viabilities compared to the control. The fold changes in cell viabilities as compared to controls were fibroblast-CM (21% O_2_): 1.22 ± 0.007 and fibroblast-CM (5% O_2_): 1.24 ± 0.001 (Figure 1(c)).

### 3.3. Increased Apoptosis with Normoxic or Hypoxic hWJSC-CM

**Figure 2:**
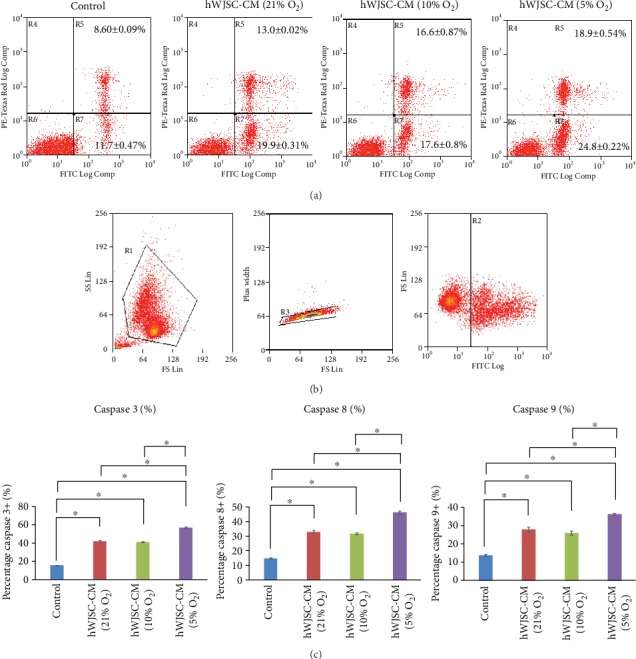
(a) Apoptotic assay (dot plots) of lymphoma cells after treatment with normoxic and hypoxic hWJSC-CM. Note significant increases in percentages of AV+PI- and AV+PI+ of lymphoma cells after treatment with hWJSC-CM as compared to the control. Treatment with hWJSC-CM collected under 5% oxygen condition showed the highest percentage increase in AV+PI- and AV+PI+ cells. (b, c) Flow cytometry dot plots and percentage caspase activity showed significant increases in caspase 3, caspase 8, and caspase 9 activities in lymphoma cells after treatment with hWJSC-CM as compared to the control. Treatment with hWJSC-CM collected under 5% oxygen conditions showed the highest increase in all caspase activities in lymphoma cells. All values are expressed as the mean ± SEM of 3 biological samples with 3 replicates for each sample. ^∗^*p* < 0.05.

The annexin V/PI assay of lymphoma cells treated with both normoxic and hypoxic hWJSC-CM showed significant increases in the percentages of early (AV+PI-) and late (AV+PI+ cells) apoptotic cells over controls. The hypoxic hWJSC-CM (5% O_2_) produced significantly higher percentages of early and late apoptotic cells as compared to normoxic hWJSC-CM (21% O_2_) and hWJSC-CM (10% O_2_). The values were hWJSC-CM (21% O_2_): 13.0 ± 0.02%, hWJSC-CM (10% O_2_): 16.6 ± 0.87%, hWJSC-CM (5% O_2_): 18.9 ± 0.54%, and control: 8.60 ± 0.09%. The percentages of late apoptotic cells were hWJSC-CM (21% O_2_): 19.9 ± 0.31%, hWJSC-CM (10% O_2_): 17.6 ± 0.80%, hWJSC-CM (5% O_2_): 24.8 ± 0.22%, and control: 11.7 ± 0.47% (Figure 2(a)).

Activated caspase 3, caspase 8, and caspase 9 activities were evaluated using flow cytometry (Figure 2(b)). Significant increases in activated caspase 3, caspase 8, and caspase 9 activities were observed in lymphoma cells as compared to controls after 48 h of treatment with hypoxic and normoxic hWJSC-CM. The hypoxic hWJSC-CM (5% O_2_) induced significantly greater increases in caspase activities as compared to normoxic hWJSC-CM (21% O_2_) and hWJSC-CM (10% O_2_) (Figure 2(c)). The percentages of lymphoma cells that were positive for activated caspase 3 were hWJSC-CM (21% O_2_): 41.7 ± 1.00%, hWJSC-CM (10% O_2_): 41.17 ± 0.68, hWJSC-CM (5% O_2_): 57.5 ± 0.26%, and control: 15.6 ± 0.40%. The percentages of lymphoma cells that were positive for activated caspase 8 activities were hWJSC-CM (21% O_2_): 32.9 ± 1.15%, hWJSC-CM (10% O_2_): 31.8 ± 0.64%, hWJSC-CM (5% O_2_): 46.2 ± 1.19%, and control: 14.8 ± 0.56%. The percentages of lymphoma cells that were positive for activated caspase 9 activities were hWJSC-CM (21% O_2_): 27.8 ± 1.37%, hWJSC-CM (10% O_2_): 26.0 ± 1.08%, hWJSC-CM (5% O_2_): 36.3 ± 0.9%, and control: 13.8 ± 0.50%.

### 3.4. Mitochondrial Superoxide, Mitochondrial Membrane Potential, and Lipid Peroxidation of Lymphoma Cells when Treated with Normoxic or Hypoxic hWJSC-CM

**Figure 3:**
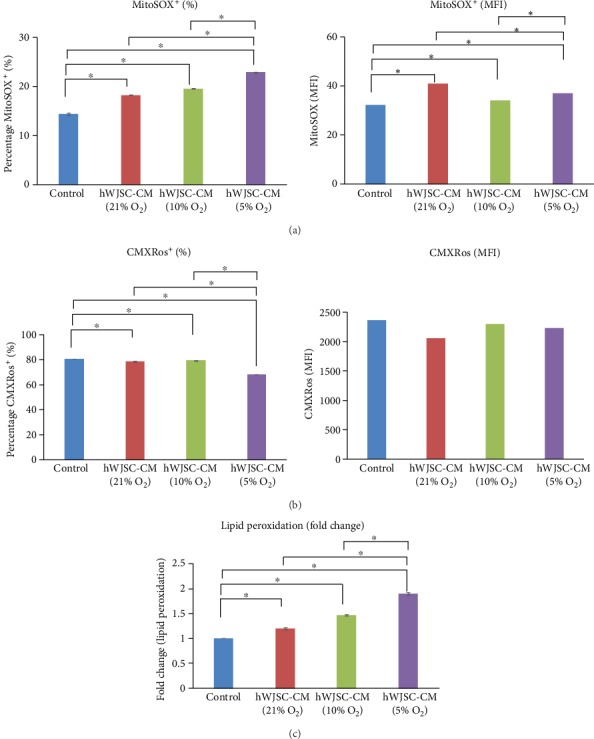
Mitochondria stress assay of lymphoma cells after treatment with normoxic and hypoxic hWJSC-CM. (a) Note significant increases in mitochondria superoxide level of lymphoma cells after treatment with hWJSC-CM as compared to control. Treatment with hWJSC-CM collected under 5% oxygen conditions showed the highest increase in lymphoma mitochondria superoxide levels. (b) Note significant decreases in mitochondria membrane potential of lymphoma cells after treatment with hWJSC-CM as compared to the control. Treatment with hWJSC-CM collected under 5% oxygen conditions showed the highest decrease in mitochondria membrane potential. (c) Note significant increases in the lipid peroxidation level of lymphoma cells after treatment with hWJSC-CM as compared to the control. Treatment with hWJSC-CM collected under 5% oxygen conditions showed the highest increase in lipid peroxidation. All values are expressed as the mean ± SEM of 3 biological samples with 3 replicates for each sample. ^∗^*p* < 0.05.

Lymphoma cells exposed to both normoxic and hypoxic hWJSC-CM showed significant increases in the number of cells positive for mitochondrial superoxide (MitoSOX) as compared to controls. The hypoxic hWJSC-CM (5% O_2_) induced significantly greater increases in the number of cells positive for MitoSOX as compared to normoxic hWJSC-CM (21% O_2_) and hypoxic hWJSC-CM (10% O_2_). The percentages of cells positive for MitoSOX were hWJSC-CM (21% O_2_): 18.3 ± 0.09%, hWJSC-CM (10% O_2_): 19.6 ± 0.05%, hWJSC-CM (5% O_2_): 22.9 ± 0.06%, and control: 14.4 ± 0.26%. The mean ± SEM fluorescence intensities of MitoSOX were hWJSC-CM (21% O_2_): 56.1 ± 1.41, hWJSC-CM (10% O_2_): 59.1 ± 1.00, hWJSC-CM (5% O_2_): 78.94 ± 0.95, and control: 42.5 ± 0.5 (Figure 3(a)).

Lymphoma cells exposed to both normoxic and hypoxic hWJSC-CM showed significant decreases in mitochondrial membrane potential (CMXRos) as compared to controls. The hypoxic hWJSC-CM (5% O_2_) induced significantly greater decreases in mitochondrial membrane potential as compared to normoxic hWJSC-CM (21% O_2_) and hypoxic hWJSC-CM (10% O_2_). The percentages of cells positive for CMXRos were hWJSC-CM (21% O_2_): 78.3 ± 0.40%, hWJSC-CM (10% O_2_): 79.3 ± 0.19%, hWJSC-CM (5% O_2_): 68.2 ± 0.05%, and control: 80.6 ± 0.02%. The mean ± SEM fluorescence intensities of CMXRos were hWJSC-CM (21% O_2_): 2050.8 ± 42.2, hWJSC-CM (10% O_2_): 2290.0 ± 44.6, hWJSC-CM (5% O_2_): 2225.5 ± 27.6, and control: 2355.3 ± 22.1 (Figure 3(b)).

Lymphoma cells treated with either normoxic or hypoxic hWJSC-CM showed significant fold change increases in lipid peroxidation as compared to controls. The hypoxic hWJSC-CM (5% O_2_) induced significantly greater fold increases in lipid peroxidation as compared to normoxic hWJSC-CM (21% O_2_) and hypoxic hWJSC-CM (10% O_2_). The fold changes of lipid peroxidation compared to controls were hWJSC-CM (21% O_2_): 1.2 ± 0.03, hWJSC-CM (10% O_2_): 1.47 ± 0.02, and hWJSC-CM (5% O_2_): 1.89 ± 0.03 (Figure 3(c)).

### 3.5. CRT, Hsp70, Hsp90, CD47, and PD-L1 Expression in Lymphoma Cells Treated with Normoxic or Hypoxic hWJSC-CM

**Figure 4:**
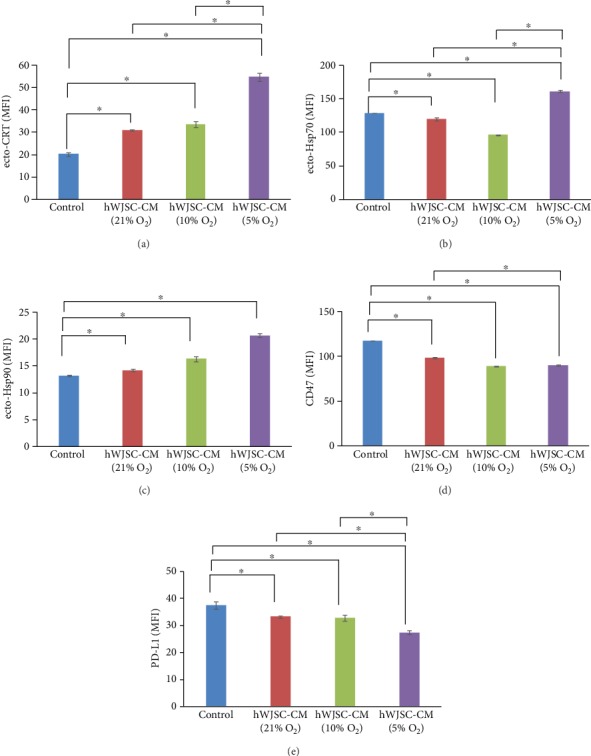
Immunogenic cell death analysis of lymphoma cells after treatment with normoxic and hypoxic hWJSC-CM. (a–c) Note significantly higher ecto-CRT, ecto-Hsp70, and ecto-Hsp90 expression after treatment with hypoxic 5% oxygen hWJSC-CM as compared to the control. (d, e) Note significantly lower CD47 and PD-L1 expressions in lymphoma cells after treatment with hWJSC-CM as compared to the control. Treatment with hWJSC-CM collected under 5% oxygen conditions showed the highest and lowest “find me/eat me” and “don't eat me” signals in lymphoma cells, respectively. All values are expressed as the mean ± SEM of 3 biological samples with 3 replicates for each sample. ^∗^*p* < 0.05.

There were significant increases in expression of surface bound ecto-CRT, ecto-Hsp70, and ecto-Hsp90 in lymphoma cells treated with normoxic or hypoxic hWJSC-CM as compared to controls. The hypoxic hWJSC-CM (5% O_2_) induced significantly greater increases in the expression of surface bound CRT, Hsp70, and Hsp90 as compared to normoxic hWJSC-CM (21% O_2_) and hypoxic hWJSC-CM (10% O_2_). The mean ± SEM fluorescence intensities of ecto-CRT were hWJSC-CM (21% O_2_): 31.0 ± 0.3, hWJSC-CM (10% O_2_): 33.8 ± 1.34, hWJSC-CM (5% O_2_): 54.8 ± 1.71, and control: 20.2 ± 0.78 (Figure 4(a)).

The mean ± SEM fluorescence intensities of ecto-Hsp70 were hWJSC-CM (21% O_2_): 9.01 ± 0.21, hWJSC-CM (10% O_2_): 8.8 ± 0.70, hWJSC-CM (5% O_2_): 9.6 ± 0.21, and control: 10.5 ± 0.30 (Figure 4(b)). The mean ± SEM fluorescence intensities of ecto-Hsp90 were hWJSC-CM (21% O_2_): 14.2 ± 0.21, hWJSC-CM (10% O_2_): 16.4 ± 0.46, hWJSC-CM (5% O_2_): 20.7 ± 0.3, and control: 13.2 ± 0.16 (Figure 4(c)).

The results showed that there were significant decreases in CD47 expression in lymphoma cells exposed to normoxic or hypoxic hWJSC-CM compared to controls. The hypoxic hWJSC-CM collected at 10% or 5% oxygen levels induced significantly greater increases in CD47 expression as compared to normoxic hWJSC-CM (21% O_2_). There were no significant differences in CD47 expression between the 10% and 5% hypoxic hWJSC-CM-treated lymphoma cells. The mean ± SEM fluorescence intensities of CD47 were hWJSC-CM (21% O_2_): 98.4 ± 1.07, hWJSC-CM (10% O_2_): 89.0 ± 0.38, hWJSC-CM (5% O_2_): 90.3 ± 0.92, and control: 117.4 ± 0.11 (Figure 4(d)).

The results showed that there were significant decreases in PD-L1 expression in lymphoma cells exposed to normoxic or hypoxic hWJSC-CM compared to controls. The hypoxic hWJSC-CM (5% O_2_) induced significantly greater decreases in PD-L1 expression as compared to normoxic hWJSC-CM (21% O_2_) and hypoxic hWJSC-CM (10% O_2_). The mean ± SEM fluorescence intensities of PD-L1 were hWJSC-CM (21% O_2_): 33.2 ± 0.48, hWJSC-CM (10% O_2_): 32.8 ± 1.10, hWJSC-CM (5% O_2_): 27.4 ± 0.61, and control: 37.5 ± 1.31 (Figure 4(e)).

### 3.6. ATP and HMGB1 Levels in Lymphoma Cells Treated with Normoxic or Hypoxic hWJSC-CM

**Figure 5:**
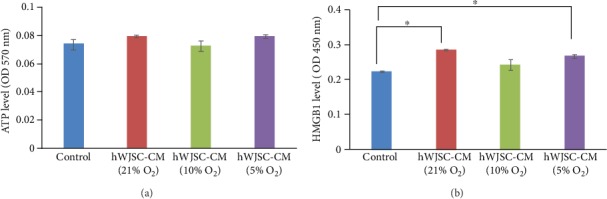
Secreted ATP and HMGB1 levels of lymphoma cells after treatment with normoxic and hypoxic hWJSC-CM. (a) Note no significant difference in extracellular ATP levels in lymphoma cells after treatment with normoxic and hypoxic hWJSC-CM as compared to the control. (b) Note significantly higher extracellular HMGB1 levels in lymphoma cells after treatment with normoxic and hypoxic 5% oxygen hWJSC-CM as compared to the control. All values are expressed as the mean ± SEM of 3 biological samples with 3 replicates for each sample. ^∗^*p* < 0.05.

The results showed that there were no significant differences in extracellular ATP levels in lymphoma cells after treatment with normoxic hWJSC-CM (21% O_2_) or hypoxic hWJSC-CM (10% O_2_ and 5% O_2_) compared to controls. The mean optical density readings measured at 570 nm for ATP levels were hWJSC-CM (21% O_2_): 0.08 ± 0.001, hWJSC-CM (10% O_2_): 0.07 ± 0.004, hWJSC-CM (5% O_2_): 0.08 ± 0.001, and control: 0.07 ± 0.004 (Figure 5(a)).

The results showed that there were significant increases in extracellular HMGB1 levels in lymphoma cells after exposure to normoxic hWJSC-CM (21% O_2_) or hypoxic hWJSC-CM (5%O_2_) compared to controls. However, there were no significant differences in extracellular HMGB1 levels observed between normoxic hWJSC-CM (21% O_2_) and hypoxic hWJSC-CM (5%O_2_). The mean optical density readings measured at 450 nm for HMGB1 levels were hWJSC-CM (21% O_2_): 0.28 ± 0.002, hWJSC-CM (10% O_2_): 0.24 ± 0.02, hWJSC-CM (5% O_2_): 0.27 ± 0.005, and control: 0.22 ± 0.003 (Figure 5(b)).

## 4. Discussion

The human umbilical cord provides for the transmission of nutrition from mother to fetus. The gelatinous nature of intervascular Wharton's jelly helps to prevent the strangulation of the umbilical blood vessels cutting off the nutritional supply to the fetus. Additionally, the unique tumoricidal properties of the stem cells lying within Wharton's jelly (hWJSCs) help to engulf and destroy any cancer cells that migrate from mother to fetus thus explaining why cancers of the fetus are extremely low in mothers who are pregnant and ill with various carcinomas [28–33].

The hWJSCs possess all the characteristics of bona fide MSCs [34], but unlike bone marrow MSCs, they do not form into cancer-associated fibroblasts in tumor environments [35–37]. hWJSCs also do not induce tumorigenesis and toxicity in laboratory animal models, cynomolgus monkeys, and in clinical trials, which suggests that they are safe when used in clinical settings [5, 38]. In almost all of these preclinical and clinical trials studied thus far, hWJSCs were maintained and cultured in normoxic conditions instead of their physiological oxygen tension levels of 5% and below. It is therefore important to evaluate whether the positive tumoricidal results obtained thus far with hWJSCs grown under normoxic conditions can be maintained or are superior when they are grown and applied under physiological hypoxic conditions.

MSCs in general including hWJSCs pass through two different phases of oxygen conditions from their *in vivo* collection, derivation *in vitro* and back to *in vivo* transplantation. Conventionally, in most culture conditions, the stem cells are usually exposed to higher oxygen concentrations during their *in vitro* manipulation compared to much lower concentrations when they are *in vivo*. Oxygen is vital in maintaining stemness, expanded growth, and differentiation of stem cells via regulation of hypoxia-inducible factor-1- (HIF1-a-) related gene expression. The culture of stem cells at a higher oxygen concentration as compared to the lower concentration in their natural niches induces environmental stress, early cell ageing, slower growth, and chromatin damage which leads to poor engraftment and suboptimal clinical results [39].

Culturing of hWJSCs at higher oxygen concentrations may have similar detrimental effects too. We observed in the present study that hWJSCs cultured under hypoxic conditions showed similar cell morphologies and could undergo trilineage differentiation into the adipocytes, osteocytes, and chondrocytes like that of normoxic hWJSC-CM (Supplemental Figure 1). Also, hWJSCs grown in hypoxic conditions (10% or 5%) remained positive for the MSC markers CD105, CD90, CD73, CD146, and CD108, and the expression levels of these markers were significantly greater than those for normoxic hWJSCs. Our results support those of other groups where there was no change in the phenotype markers when cultured in hypoxic or normoxic conditions [40, 41].

Although our results showed that normoxic and hypoxic hWJSC-CM induced significant reduction in lymphoma cell viability and showed increased numbers of apoptotic cells, the exposure to hypoxic hWJSC-CM (5% O_2_) showed the greatest cell viability reduction and apoptotic cell numbers. These tumoricidal effects are consistent with previous published studies where hypoxic WJSC-CM exhibited greater attenuation of cell growth of cervical, liver, prostate, and ovarian cancers as compared to normoxic WJSC-CM [40, 41]. We also observed that treatment of lymphoma cells with hypoxic hWJSC-CM induced the highest oxidative stress in the mitochondria and with the highest levels of lipid peroxidation as compared to normoxic hWJSC-CM. The presentation of “find-me/eat-me”-danger-associated molecular pattern (DAMP) molecules and the reduced expression of the “don't eat me” signals (PD-L1 and CD47) in the dying cancer cells trigger an immune response by the host and achieve complete remission [19, 20]. There is growing evidence that anticancer agents that could stimulate tumor-specific responses via immunogenic cell death improved the efficacy and long-term therapeutic success [42]. Likewise, strategies to block CD47 and PD-L1 expressions on cancer cells are of immense interest as they enhance the antitumor response [19, 43]. Our study showed that treatment with hypoxic hWJSC-CM at 5% oxygen tensions had the greatest upregulation of the DAMPs such as ecto-CRT, ecto-Hsp70, Hsp-90, and HMGB1 and downregulation of CD47 and PD-L1. We hypothesize that culturing hWJSCs and collection of hWJSC-CM in normoxic and 10% hypoxic conditions may not produce differences in the various types of factors (secretome) in the hWJSC-CM. As such, we did not see significant differences in the anticancer effect between normoxic hWJSC-CM and 10% oxygen hWJSC-CM. The differences in the anticancer effect were observed when we cultured hWJSCs and collected hWJSC-CM in 5% hypoxic conditions. This coincides with the literature that suggests that the physiological oxygen tension in the umbilical cord is about 5% (between 2% and 8%).

The results suggest that using 5% hypoxic hWJSC-CM will elicit a greater antitumor immunity response as compared to hWJSC-CM prepared under the typical normoxic culture conditions.

The increased tumoricidal effect of hWJSC-CM prepared under hypoxic conditions may be due to increased expression and secretion of anticancer molecules or microvesicles/exosomes as compared to hWJSC-CM prepared under normoxic conditions. Expression of HIF1-a was also previously shown to be upregulated in hypoxic conditions and in turn regulate many downstream process stemness and differentiation of stem cells [39]. We have previously reported that hWJSC-CM contains significantly higher levels of interleukins and adhesion molecules like IL-1a, IL-6, IL-8, HGF, SCF, and MCP-1 that regulate cancer cell death and modulate the immune system [17]. We have also reported that hWJSCs highly express miRNA-146a and miRNA-126 that target the innate and adaptive immune system [44]. The expression of miRNA-146a and miRNA-126 has been reported to activate the T cell immunity or prevent the growth of various cancer cells [45–53]. Further studies aimed at comparatively evaluating the hypoxic and normoxic hWJSC-CM secretome and HIF1-a pathways will give further insights on the various molecules involved in the increased tumoricidal and immunogenic cell death observed with hypoxic hWJSC-CM which can eventually be characterized and tested for their tumoricidal effects singly or in combinations.

Though this study evaluated the differences in tumoricidal effects of hWJSCs cultured under different oxygen concentrations, there is a lack of information on the exposure of hWJSCs to atmospheric oxygen concentrations during the isolation or derivation process. It has been demonstrated that brief exposure to ambient oxygen concentrations during the collection and isolation has rapid and irreversible changes to the metabolism in cord blood hematopoietic stem cells or progenitor cells (HSC/HPC) [54]. The cells undergo extra physiologic oxygen shock/stress (EPHOSS) where they produce ambient air-induced mitochondrial ROS which initiate the opening of the mitochondrial permeability transition pore [55]. Collection, processing, and culturing of HSC/HPCs under hypoxic conditions enhanced the collection and proliferation of long-term repopulating HSCs. However, the collection and processing at low oxygen tensions are a huge logistical problem that is both cumbersome and expensive. The addition of cyclosporin A, an FDA-approved drug, during the collection and processing of cord blood has been shown to mitigate the effect of EPHOSS by mimicking the effects of low oxygen concentration [54]. As most of the EPHOSS effect studies were on HSCs/HPCs [54], there is limited information on the EPHOSS on MSCs and hWJSCs. More studies are needed to evaluate the metabolism of hWJSCs affected by EPHOSS and whether the addition of cyclosporin A into the processes could generate hWJSCs with higher therapeutic potential.

## 5. Conclusion

Our results suggest that hypoxic hWJSC-CM induces a greater tumoricidal response via the presentation of DAMPs to initiate antitumor immunity. Hypoxic hWJSC-CM could also suppress the defense molecule expression on lymphoma cells that could make the lymphoma cells more prone to an immune response by the host in order to achieve complete remission. Although both normoxic and hypoxic hWJSC-CM hold tremendous promise as novel agents against malignant tumors, hypoxic hWJSC-CM should be the agent of choice because of its greater tumoricidal effects.

## Figures and Tables

**Table 1:** Flow cytometric analysis of hWJSC cell surface markers cultured in 21% oxygen, 10% oxygen, and 5% oxygen.

Surface markers	21% oxygen	10% oxygen	5% oxygen	*p* value (one-way ANOVA)
MSC markers (percentage (%))				
CD73	98.66 ± 0.08	98.75 ± 0.10	96.34 ± 0.29	n.s.
CD90	98.59 ± 0.04	98.44 ± 0.12	94.83 ± 0.14	n.s.
CD105	95.76 ± 0.06	97.93 ± 0.06	95.43 ± 0.15	n.s.
CD108	96.46 ± 0.02	96.90 ± 0.01	93.20 ± 0.01	n.s.
CD146	94.86 ± 0.07	96.42 ± 0.15	95.57 ± 0.10	n.s.
HSC markers (percentage (%))				
CD14	3.02 ± 0.05	2.33 ± 0.09	4.26 ± 0.01	n.s.
CD19	2.01 ± 0.02	2.15 ± 0.10	3.55 ± 0.12	n.s.
CD34	1.91 ± 0.01	1.92 ± 0.06	4.39 ± 0.04	n.s.
CD45	2.35 ± 0.14	2.32 ± 0.15	3.34 ± 0.06	n.s.
CD117	1.81 ± 0.07	2.39 ± 0.08	2.74 ± 0.06	n.s.

Results are presented as the mean ± SEM (%). n.s.: not significant.

**Table 2:** Flow cytometric analysis of hWJSC cell surface marker expression level (MFI) cultured in 21% oxygen, 10% oxygen, and 5% oxygen.

Surface markers	21% oxygen	10% oxygen	5% oxygen	*p* value (one-way ANOVA)
MSC marker expression (MFI)				
CD73	90.33 ± 0.16	105.46 ± 1.22	162.36 ± 0.49	*p* < 0.05 (21% vs. 10% vs. 5%)
CD90	103.23 ± 1.32	186.90 ± 1.16	313.82 ± 1.57	*p* < 0.05 (21% vs. 10% vs. 5%)
CD105	17.32 ± 0.46	35.35 ± 0.05	58.59 ± 0.48	*p* < 0.05 (21% vs. 10% vs. 5%)
CD108	81.29 ± 0.24	74.35 ± 0.57	188.28 ± 1.18	*p* < 0.05 (21% vs. 10% vs. 5%)
CD146	44.86 ± 5.02*E* − 15	63.09 ± 0.11	130.16 ± 0.19	*p* < 0.05 (21% vs. 10% vs. 5%)
HSC marker expression (MFI)				
CD14	4.26 ± 1.014	4.66 ± 0.25	4.64 ± 0.12	n.s.
CD19	3.14 ± 0.27	3.46 ± 0.01	4.29 ± 0.17	n.s.
CD34	3.91 ± 0.46	3.46 ± 0.01	8.42 ± 0.27	*p* < 0.05 (21% vs. 5%)
CD45	2.72 ± 0.26	4.49 ± 0.57	3.57 ± 0.21	n.s.
CD117	3.21 ± 0.02	4.32 ± 0.13	3.84 ± 0.06	*p* < 0.05 (21% vs. 10%)

Results are represented as the mean ± SEM (%). n.s.: not significant.

## Data Availability

The data used to support the findings of this study are included within the article.

## Supplementary Materials

Supplementary Materials Morphology and trilineage differentiation of hWJSCs cultured under normoxic (21% O_2_) and hypoxic (10% or 5%) conditions. (A) hWJSCs cultured in normoxic and hypoxic conditions were plastic adherent and showed no distinct morphological difference between them. (B) Both normoxic and hypoxic hWJSCs differentiated into adipocytes after 21 days in adipogenesis differentiation medium. The normoxic and hypoxic hWJSCs exhibited lipid droplets with Oil Red O staining. (C) Both normoxic and hypoxic hWJSCs differentiated into osteocytes after 21 days in osteogenic differentiation medium. The normoxic and hypoxic hWJSCs exhibited calcium deposition with Von Kossa staining. (D) Both normoxic and hypoxic hWJSCs differentiated into chondrocytes after 21 days in chondrogenic differentiation medium. The normoxic and hypoxic hWJSCs exhibited glycosaminoglycans with Alcian blue staining.
