# Supplementary material for: Hypoxic Wharton's Jelly Stem Cell Conditioned Medium Induces Immunogenic Cell Death in Lymphoma Cells
Source: Stem Cells Int. 2020 Apr 20;2020:4670948. doi: 10.1155/2020/4670948 (PMC7189315; doi:10.1155/2020/4670948)
Supplement: Supplementary Materials — Morphology and trilineage differentiation of hWJSCs cultured under normoxic (21% O2) and hypoxic (10% or 5%) conditions. (A) hWJSCs cultured in normoxic and hypoxic conditions were plastic adherent and showed no distinct morphological difference between them. (B) Both normoxic and hypoxic hWJSCs differentiated into adipocytes after 21 days in adipogenesis differentiation medium. The normoxic and hypoxic hWJSCs exhibited lipid droplets with Oil Red O staining. (C) Both normoxic and hypoxic hWJSCs differentiated into osteocytes after 21 days in osteogenic differentiation medium. The normoxic and hypoxic hWJSCs exhibited calcium deposition with Von Kossa staining. (D) Both normoxic and hypoxic hWJSCs differentiated into chondrocytes after 21 days in chondrogenic differentiation medium. The normoxic and hypoxic hWJSCs exhibited glycosaminoglycans with Alcian blue staining. [file 4670948.f1.docx]

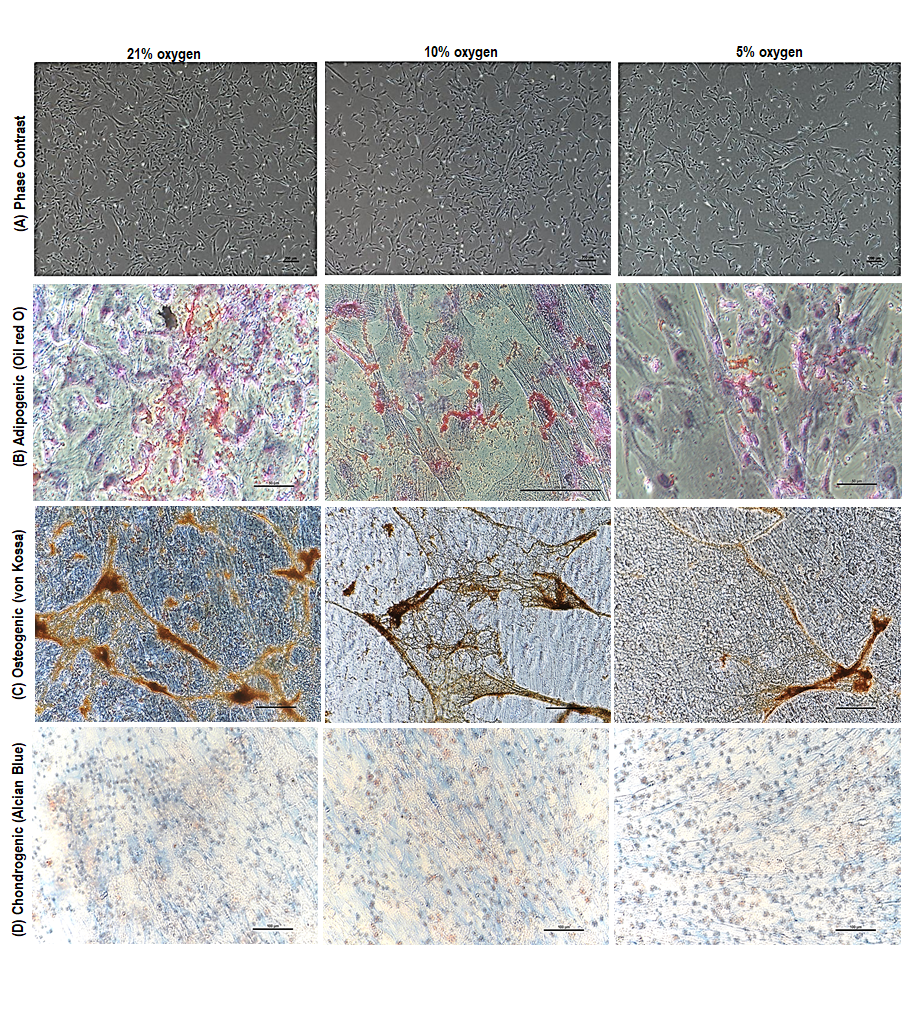
**Supplementary Figure 1 Morphology and tri-lineage differentiation of hWJSCs cultured under normoxic (21% O_2_) and hypoxic (10% or 5%) conditions**. (A) hWJSCs cultured in noromoxic and hypoxic conditions were plastic adherent and showed no distinct morphological difference between them. (B) Both normoxic and hypoxic hWJSCs differentiated into adipocytes after 21 days in adipogenesis differentiation medium. The normoxic and hypoxic hWJSCs exhibited lipid droplets with Oil Red O staining. (C) Both normoxic and hypoxic hWJSCs differentiated into osteocytes after 21 days in osteogenic differentiation medium. The normoxic and hypoxic hWJSCs exhibited calcium deposition with von Kossa staining. (D) Both normoxic and hypoxic hWJSCs differentiated into chondrocytes after 21 days in chondrogenic differentiation medium. The normoxic and hypoxic hWJSCs exhibited glycosaminoglycans with Alcian blue staining.
